# The time course of auditory looming cues in redirecting visuo-spatial attention

**DOI:** 10.1038/s41598-018-36033-8

**Published:** 2019-01-24

**Authors:** Christiane Glatz, Lewis L. Chuang

**Affiliations:** 1 Max Planck Institute for Biological Cybernetics, Department Human Perception, Cognition, and Action, Tübingen, 72076 Germany; 2 Graduate Training Centre of Neuroscience, University of Tübingen, Tübingen, 72074 Germany; 3 Institute for Informatics, Ludwig-Maximilian-Universiät, Munich, 80337 Germany

## Abstract

By orienting attention, auditory cues can improve the discrimination of spatially congruent visual targets. Looming sounds that increase in intensity are processed preferentially by the brain. Thus, we investigated whether auditory looming cues can orient visuo-spatial attention more effectively than static and receding sounds. Specifically, different auditory cues could redirect attention away from a continuous central visuo-motor tracking task to peripheral visual targets that appeared occasionally. To investigate the time course of crossmodal cuing, Experiment 1 presented visual targets at different time-points across a 500 ms auditory cue’s presentation. No benefits were found for simultaneous audio-visual cue-target presentation. The largest crossmodal benefit occurred at early cue-target asynchrony onsets (i.e., CTOA = 250 ms), regardless of auditory cue type, which diminished at CTOA = 500 ms for static and receding cues. However, auditory looming cues showed a late crossmodal cuing benefit at CTOA = 500 ms. Experiment 2 showed that this late auditory looming cue benefit was independent of the cue’s intensity when the visual target appeared. Thus, we conclude that the late crossmodal benefit throughout an auditory looming cue’s presentation is due to its increasing intensity profile. The neural basis for this benefit and its ecological implications are discussed.

## Introduction

Whilst driving a car, we have to concentrate on the road ahead while remaining alert to sudden events in our visual periphery, such as the sudden appearance of a jaywalking pedestrian. Fortunately, we are well-equipped to deal with the abrupt appearance of such critical events. They involuntarily and rapidly attract attention to themselves^1^ and confer faster and more accurate processing to spatially coincident events that follow thereafter^2–7^. Reflexive orienting can occur crossmodally. Like visual events, auditory events can similarly capture visuo-spatial attention and confer cuing benefits to visual targets within spatial proximity^8–10^. However, this involuntary orienting of spatial attention is transient and is not sustained indefinitely. Especially, if attention is required elsewhere—such as, returning to our original example, the road ahead. It is well-established that the time course of orienting occurs and decays rapidly^2,11,12^. In fact, observable benefits of cuing start to reverse when cues and targets are separated by durations longer than 300 ms^13–15^. These known properties of visuo-spatial attention raise some interesting questions. First, to what extent will an auditory cue reorient and maintain visuo-spatial attention throughout its own presentation? To date, most studies on crossmodal spatial orienting have employed auditory cues with short durations (e.g., 83–250 ms)^8,9,16^. Nonetheless, auditory events (e.g., speech, objects) are typically characterized by how they change over time. Even simple changes in intensity could signal whether an object is approaching or departing. Thus, could spatiotemporal changes in an auditory cue also influence the time course by which it redirects visuo-spatial attention? The current work investigates how changes in an auditory cue’s intensity profile might influence its ability to redirect visuo-spatial attention. Specifically, we report two experiments that demonstrate that auditory looming cues (i.e., sounds with rising intensities) are able to redirect and sustain visuo-spatial attention until the end of their presentation (Experiment 1), in a way that does not depend on their intensity levels per se (Experiment 2). In contrast, auditory cues with decreasing or steady-state intensity profiles elicit a rapid deployment of transient attention that does not last throughout their presentation.

Objects appear to approach us when they expand visually or when they get louder with time. Such objects, termed *looming*, are claimed to be especially salient because they signal imminent threats. For example, visual looming objects induce involuntary fear and avoidance responses in mice^17^, rhesus monkeys^18^, and human infants^19^, which suggests reflexive and innate mechanisms to looming stimuli. The auditory equivalent, namely looming sounds with rising intensities, have also been associated with preferential processing and alerting responses. Looming sounds elicit larger skin conductance responses^20^ and amygdala activity^21^ than receding sounds (but see^22^ for an alternative account in the context of auditory motion in music). Looming sounds with rising intensities are often perceived as changing more than their equivalent receding counterparts with falling intensities^21,23–26^. Moreover, looming sounds are also perceived as having a longer duration than their receding equivalent^27–31^, which indicate that they might be attended to for longer durations. Finally, looming sounds are associated with greater activity in the auditory cortex, as well as in neural networks related to attention and spatial processing^32–34^. Taken together, it is generally agreed that looming sounds are salient auditory stimuli that increase phasic alertness, presumably because they communicate approaching threats^24^.

The saliency of looming sounds can influence visual perception. For example, static visual targets are perceived as larger or brighter than they really are, when accompanied by looming sounds^35^. In a more realistic setting, drivers braked earlier if a potential head-on collision was accompanied with a looming sound, relative to a static auditory warning^36^. More interestingly, auditory looming stimuli can induce excitation in visual cortical regions for low-level processing^37,38^. These interactions are often discussed in terms of multisensory integration^38,39^. Nonetheless, there is some evidence that looming sounds can also exert a preferential bias on visuo-spatial attention. When presented in only one ear, a looming sound can increase tilt discrimination sensitivity in the congruent visual hemifield relative to the opposing hemifield, for an object that is presented simultaneously^16^. This raises the question: What is the role (if any) of a looming sound in reorienting visuo-spatial attention?

Looming sounds are salient events that can enhance the perception of visual targets^37^, especially targets that are spatially congruent^16^. In the current study, we investigate whether auditory looming cues might exert a crossmodal influence on visuo-spatial attention, across its presentation duration, in a way that might differ from other similar auditory cues. In particular, we contrast looming sounds against sounds with a steady-state intensity or a decreasing intensity profile. If looming sounds are salient and exert a strong bottom-up influence on reorienting visuo-spatial attention, we expect cuing benefits to be larger at short cue-target onset asynchronies (CTOAs; i.e., 250 ms) compared to other auditory cues. Independent of cuing benefits, auditory looming sounds might even improve the discrimination of simultaneously presented visual targets (cf., Leo *et al*.^16^), given that they are known to generally facilitate visual processing^37,38^. In both cases, such cuing benefits of reflexive visuo-spatial attention can be expected to diminish with time and, potentially, throughout the presentation of the sounds themselves. On the other hand, the ongoing presentation of looming sounds (but not static and receding sounds) might indicate the continuous approach of a visual target, motivating participants to not only redirect but to sustain their attention^40^. This would result in cuing benefits that last until the end of the auditory cue’s presentation.

Experiment 1 varied the CTOAs between different auditory cues and the visual target of a peripheral tilt-discrimination task. However, it has to be noted that looming cues were physically louder than the static and receding cues at the end of their presentation. Furthermore, previous studies have shown that looming sounds also tend to be perceived as louder^41–43^ and longer-lasting^27–31^ than their receding equivalents, perhaps due to a recency effect^25,26^. Given that the looming cue benefit at 500 ms CTOA could have resulted from the intensity differences between the auditory cues at 500 ms, Experiment 2 manipulated the final intensity levels of static and looming cues and compared their cuing benefits at a fixed CTOA of 500 ms. Experiment 2 verified that the cuing benefit of auditory looming cues, unlike auditory static cues, was independent of their respective intensities when the visual target appeared. Two key aspects set the current study apart from previous research. Unlike most studies on spatial orienting, we employed a dual-task paradigm that required participants to perform a central manual tracking task at all times. In other words, diverting spatial attention away from the central location comes at a cost, which can reasonably be assumed to be larger than if participants were merely requested to maintain central fixation. Another distinction is the use of looming sounds as a spatially valid cue to direct attention to the location of an upcoming target.

The current experiments were designed to examine how sounds govern visuo-spatial orienting throughout their presentation. Hence, we employed auditory cues with durations that were longer than comparable audio-visual crossmodal cuing studies^8,9^ and presented visual targets at different points across their presentation duration. In this regard, our experiment design differs from previous work that have similarly addressed the crossmodal influence of looming sounds on visual processing. To begin, previous studies have typically presented a visual target simultaneously with the onset of a looming sound^16,38,39,44,45^ or after a looming sound has been presented^20,21^. The former paradigm typically addressed supramodal influences of looming sounds on multisensory integration and the latter, aspects related to phasic alerting. Few studies^16^ have directly investigated how looming sounds influence visuo-spatial attention. Therefore, the current study is the first to describe the time course of an auditory cue’s crossmodal influence on orienting visuo-spatial attention.

## Experiment 1: Do auditory looming sounds enhance peripheral tilt-discrimination performance across its presented duration?

### Results and Discussion

**Figure 1:**
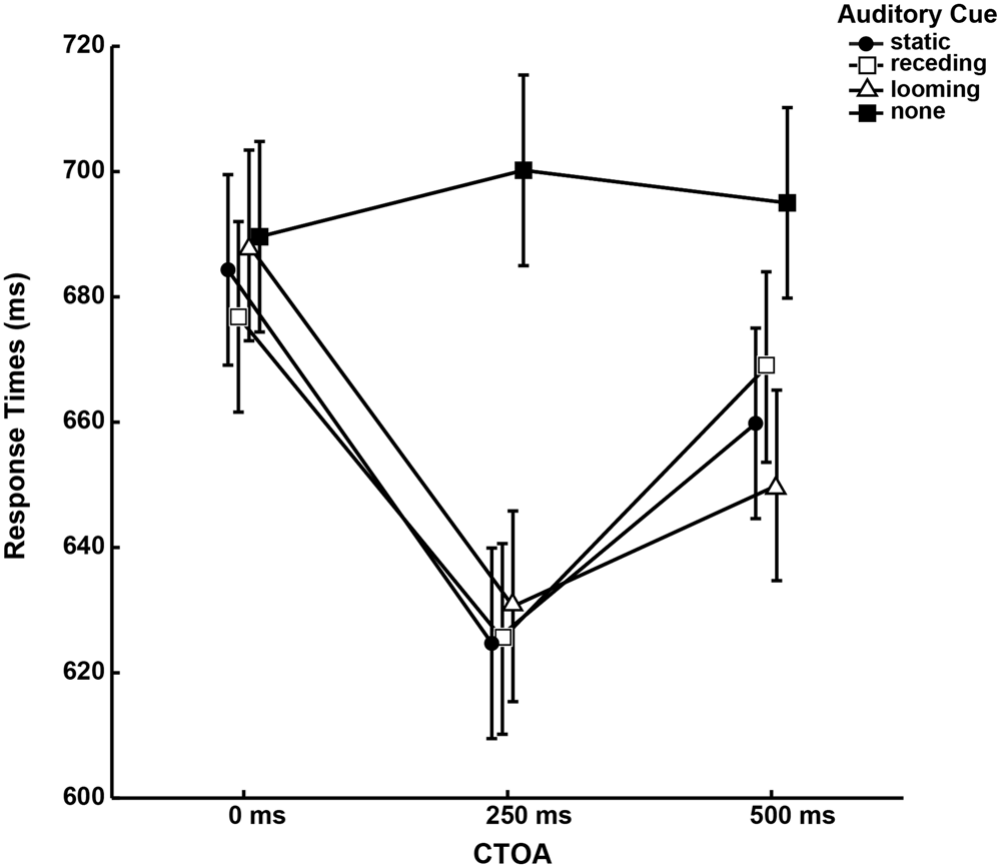
Interaction of *Auditory Cue* and *CTOA*. Cued reaction times are fastest for the CTOA level of 250 ms, relative to the simultaneous presentation (0 ms) of cue and target. This reaction time benefit decreases at 500 ms, particularly for static and receding cues. Error bars represent 95% confidence intervals according to^70^.

Performance in the peripheral tilt-discrimination task was operationalized in terms of the time that a participant took to respond correctly from the time of target appearance (RTs). Participants responded on average 72.7% of the times correct. To compensate for positive skews in RT measures^46^, medians RTs were calculated for each experimental condition. This data is presented in Fig. 1. There is a general pattern of cuing benefits, irrespective of auditory cue types, that peaks for visual targets that appear 250 ms after the onset of the auditory cue and diminish for those that appear 500 ms after cue onset. Interestingly, there appears to be no benefit for the discrimination of visual targets that appear simultaneously with the auditory cues.

The median RTs were submitted to a repeated measures ANOVA (JASP^47^; see Supplementary Material) for the factors of *Auditory Cue* (none, looming, receding, static) and *CTOA* (0, 250, 500 ms). There was significant interaction between the factors of *Auditory Cue* and *CTOA* (*F*(6, 84) = 13.383, *p* = 0.001, ω^2^ = 0.449) as well as for both main effects (*Auditory Cue*: (*F*(3, 42) = 20.553, *p* = 0.001, ω^2^ = 0.560); *CTOA*: (*F*(1.374, 19.242) = 9.155, *p* = 0.004, ω^2^ = 0.345). To interpret the interaction, we performed separate one-way ANOVAs for the factor of *CTOA* for each auditory cue condition. With the exception of the ‘none’ condition, all conditions returned a significant main effect for *CTOA*. For auditory static cues, significantly faster RTs were found at a CTOA of 250 ms, compared to 0 ms (*t*(14) = 4.746, *p*_*bonf*_ <0.001, *d* = 1.226) and 500 ms (*t*(14) = 2.798, *p*_*bonf*_ = 0.028, *d* = 0.722). Auditory receding cues showed a similar pattern of faster RTs at a CTOA of 250 ms, compared to 0 ms (*t*(14) = 5.152, *p*_*bonf*_ <0.001, *d* = 1.330) and 500 ms (*t*(14) = 4.354, *p*_*bonf*_ <0.001, *d* = 1.124). The RTs between CTOAs of 0 ms and 500 ms neither differed for auditory static cues (*t*(14) = 1.949, *p*_*bonf*_ = 0.184, *d* = 0.503) nor auditory receding cues (*t*(14) = 0.798, *p*_*bonf*_ = 1.000, *d* = 0.206). In contrast, auditory looming cues showed a different pattern of RTs across CTOA levels. Compared to a CTOA of 0 ms, RTs were significantly faster at both CTOAs of 250 ms (*t*(14) = 4.471, *p*_*bonf*_ <0.001, *d* = 1.154) and 500 ms (*t*(14) = 2.971, *p*_*bonf*_ =0.018, *d* = 0.767). Interestingly, RTs did not differ significantly between CTOAs of 250 ms and 500 ms (*t*(14) = 1.500, *p*_*bonf*_ =0.434, *d* = 0.387). Finally, we contrasted the median RTs of the three cue conditions at the CTOA level of 500 ms with two-tailed paired-samples t-tests. Looming cues gave rise to faster RTs at 500 ms than receding cues (*t*(14) = 2.281, *p* = 0.039, *d* = 0.589). There were no significant differences between static and looming cues (*t*(14) = 0.965, *p* = 0.351, *d* = 0.249), and static and receding cues (*t*(14) = 1.199, *p* = 0.250, *d* = 0.310).

Discrimination sensitivity^48^ (*d*′) were submitted to the same repeated measures ANOVA to determine if there were speed-accuracy tradeoffs across the cued conditions. There were no significant main effects for *Auditory Cue* (*F*(3, 42) = 0.304, *p* = 0.823, ω^2^ = 0.000) and *CTOA* (*F*(2, 28) = 0.023, *p* = 0.372, ω^2^ = 0.002). There was also no significant interaction for *Auditory Cue* and *CTOA* (*F*(6, 84) = 0.739, *p* = 0.620, ω^2^ = 0.000).

The root-mean-squared-error (RMSE) of manual tracking during auditory cue presentation was also evaluated to determine if the cues impaired central task performance. There were no significant main effects of *Auditory Cue* (*F*(3, 42) = 0.938, *p* = 0.431, ω^2^ = 0.000), *CTOA* (*F*(2, 28) = 0.533, *p* = 0.539, ω^2^ = 0.000), or their interaction (*F*(6, 84) = 1.393, *p* = 0.227, ω^2^ = 0.025).

The results of Experiment 1 reveal that auditory cues can induce a crossmodal reorienting of spatial attention that result in faster tilt-discriminations of peripheral targets. In a dual-task paradigm with a central task that demands attention continuously, this manifests itself as a response time benefit, with no influence on discrimination sensitivity and at no noticeable cost to central task performance. Generally, this crossmodal benefit is transient. It peaks when the visual targets appear shortly after auditory cue onset at a CTOA of 250 ms and diminishes with extended cue presentation at a CTOA of 500 ms. Contrary to our expectations, auditory looming cues did not exhibit cuing benefits that were generally stronger compared to the static or receding cue. At 250 ms CTOA, all auditory cues resulted in comparable cuing benefits relative to the simultaneous presentation of auditory cue and visual target at CTOA 0 ms. Interestingly, there continued to be a cuing benefit for auditory looming cues at a CTOA of 500 ms that significantly differed from CTOA 0 ms, but not from CTOA 250 ms. This pattern of results was not observed for the static and receding cued trials that demonstrated a cuing benefit only at CTOA 250 ms. Hence, we propose that looming cues result in a cuing benefit that does not diminish as readily as static and receding cues. Nonetheless, these results could also be explained by the intensity of auditory cues at visual target onset. A comparison of the RTs at 500 ms CTOA reveal a significant difference between the loudest (i.e., looming) and softest (i.e., receding) cues, but not between these extremes and the cue with intermediate intensity (i.e., static). Regardless of either explanation, the current results eliminate the possibility that changes in auditory intensity over time (i.e., auditory motion) can, in themselves, result in preferential cuing benefits. The next experiment seeks to clarify if cue benefits at long CTOA (i.e., 500 ms) ought to be attributed to cue intensity or their looming characteristic.

## Experiment 2: Can the sustained performance benefit of a looming sound at late CTOAs be attributed to its high intensity when the visual target appears?

Experiment 1 demonstrated that looming sounds exert a significant crossmodal cue benefit to visual targets that subsequently appeared at either CTOAs 250 ms and 500 ms, compared to when they appeared simultaneously with the visual target (i.e., CTOA 0 ms). In contrast, the crossmodal cue benefit for receding and static sounds was only significant at CTOA 250 ms but not at 500 ms, compared to simultaneous presentation with the visual target. Taken together, this suggests that looming sounds exert a similar reorienting effect as other sounds (i.e., 250 ms), but unlike other sounds maintains this attention for longer. Nonetheless, a direct comparison of the cue benefits at CTOA 500 ms suggested that the final intensity (i.e., loudness) of the sounds could have played a supplementary role in maintaining attention at long CTOAs. Specifically, the late crossmodal cue benefit (i.e., CTOA 500 ms) of looming sounds was significantly larger than the receding sound, though not in comparison to static sound whose cue benefit did not differ from either the receding or looming sounds.

**Figure 2:**
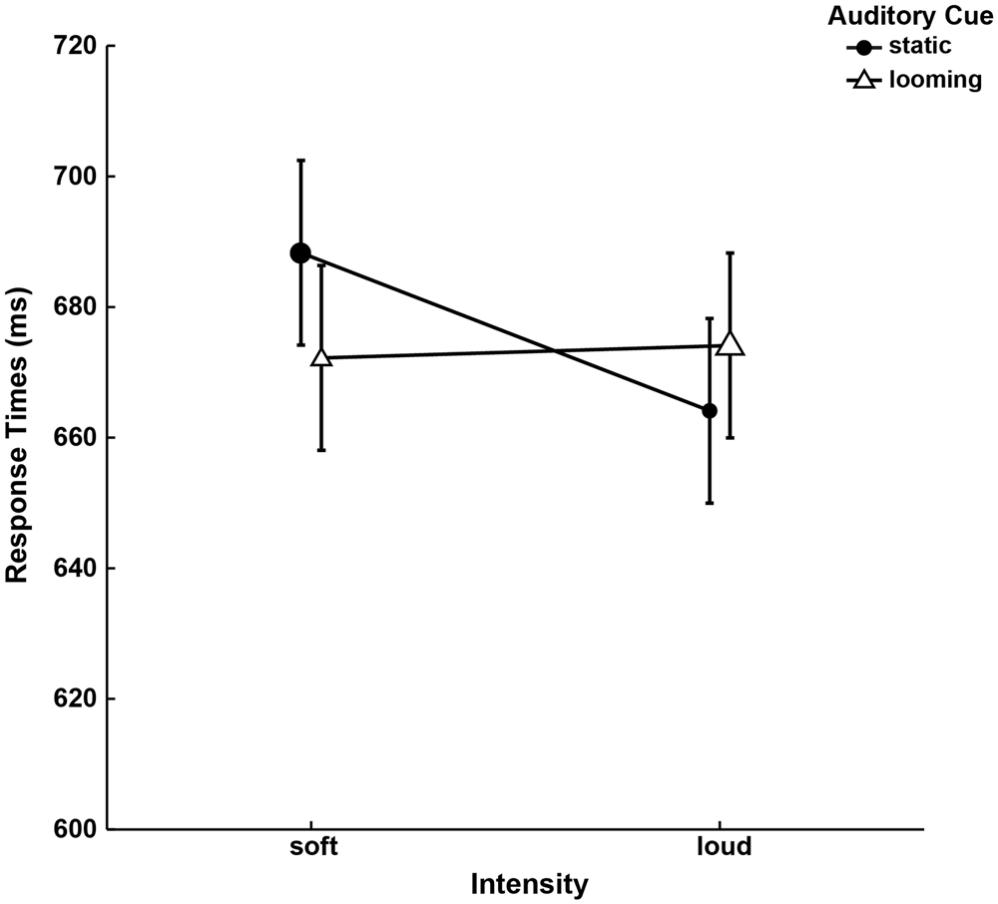
Interaction of *Auditory Cue* and *Intensity*. Median RTs of looming cues do not vary with *Intensity* levels. In contrast, loud static cues induce faster RTs than soft static cues. The conditions indicated by larger icons were identical to the static and looming conditions in Experiment 1. Error bars represent 95% confidence intervals according to^70^.

Experiment 2 was designed to discriminate between the influence of sound intensity and cue type on late crossmodal cue benefits (i.e., CTOA 500 ms). Therefore, we varied for the independent variables of *Intensity* (soft, loud) and *Auditory Cue* (static, looming). The soft-static and loud-looming cues were identical to the static and looming sounds of Experiment 1. Their cue benefits are represented in Fig. 2 with larger icons. There were two new auditory cues. The steady-state intensity of the loud-static cue was equivalent to the offset intensity of the original looming sound. The offset intensity of the soft-looming cue was equivalent to the steady-state intensity of the original static sound. To reiterate, Experiment 2 was designed to determine if the late crossmodal cue benefits exhibited by looming sounds were due to their final intensity levels.

### Results and Discussion

To begin, we performed one-tailed paired samples t-tests (α = 0.05) and confirmed that all auditory cues induced a late crossmodal cue benefit on visual targets (range of means = 664–688 ms), compared to instances when targets were not preceded by an auditory cue (mean = 736 ms; SE = 79). Figure 2 summarizes the RTs across the conditions that presented an auditory cue. Auditory looming cues appear to induce a cuing benefit that does not change with intensity levels, while a loud-static cue induces a larger cuing benefit than a soft-static cue.

The median RTs of Experiment 2 (Fig. 2) were submitted to a repeated-measures ANOVA for the factors of *Auditory Cue* (static, looming) and *Intensity* (soft, loud). There were no significant main effects of *Auditory Cue* (*F*(1, 14) = 0.230, *p* = 0.639, ω^2^ = 0.000) and *Intensity* (*F*(1, 14) = 1.486, *p* = 0.243, ω^2^ = 0.029). More importantly, this analysis revealed a significant interaction (*F*(1, 14) = 7.305, *p* = 0.017, ω^2^ = 0.000), confirming our interpretation of the cuing benefits of the different auditory cues.

The soft-static cues and loud-looming cues were respectively equivalent to the static and looming cues employed in Experiment 1. Planned two-tailed paired-samples t-tests revealed a significant difference between the cuing benefits of soft-static and soft-looming (*t*(14) = 2.220, *p* = 0.043, d = 0.573), and no significant difference between loud-looming and loud-static cues (*t*(14) = 1.156, *p* = 0.267, d = 0.298). In other words, the looming sounds confer a late crossmodal cue benefit regardless of their intensity levels. In contrast, the late crossmodal cue benefit of static cues appear to be depend on their intensity levels.

The same analyses were performed on *d*′ scores. Paired samples t-tests confirmed that none of the auditory cues improved discrimination sensitivity (range of means = 1.680–1.781) relative to uncued trials (mean = 1.653, SE = 0.206). The ANOVA revealed no significant main effects and interactions for the factors of *Auditory Cue* and *Intensity*.

Similarly, we analyzed the RMSE on the visuo-motor tracking task to check for interference from auditory cues. Paired samples t-tests confirmed that none of the auditory cues significantly reduced central task performance (range of means = 1.374–1.438), relative to comparable periods when no cue was presented (mean = 1.407, SE = 0.082). The ANOVA revealed no significant main effects and interactions for the factors of *Auditory Cue* and *Intensity*.

To summarize, we found that auditory looming cues confer similar magnitudes of late crossmodal cue benefits at the end of their presentation. In other words, Experiment 1′s results cannot be solely attributed to the looming cue’s loudness levels when the visual target appeared.

## Discussion

The current results reveal that looming sounds attract visuo-spatial attention to their locations and confer, regardless of their loudness, an early and late crossmodal cue benefit. In contrast, comparable sounds (i.e., static and receding sounds) introduce an early cue benefit that wanes, even during their presentation. Although auditory cue intensity could influence the magnitude of cue benefits over time, this did not appear to be the reason for looming sound’s persistent cue benefit. In fact, a looming sound that ends loudly elicits a cue benefit that is indistinguishable from a looming sound that ends softly. In contrast, the cue benefit of static sounds is highly dependent on the cues steady-state intensities. Hence, we believe that looming sounds exert a unique crossmodal influence on visuo-spatial attention; they draw visuo-spatial attention to themselves as well as compel it to remain for the entirety of their presentation. This holds, even when visuo-spatial attention is demanded elsewhere—that is, in our study, a central manual tracking task.

At first glance, our results differ from those reported by Leo and colleagues^16^. To recap, Leo *et al*.^16^ reported that monoaural auditory looming cues induce a preferential bias in tilt-discrimination sensitivity of the visual hemifield that is spatially congruent to the presentation ear, relative to the hemifield that is incongruent. However, we only found RT benefits when the auditory cues preceded visual targets and not when they appeared simultaneously with the visual targets (i.e., 0 ms CTOA). Several differences exist between our experiments. Unlike Leo and colleagues, we sought to investigate how a crossmodal influence of auditory cues on spatially congruent visual targets might operate throughout their presentation. Therefore, we varied the CTOAs of auditory cues and peripheral visual targets, without varying spatial congruency. In contrast, Leo *et al*.^16^ evaluated the crossmodal influence of non-predictive sounds on simultaneously presented visual targets, which were either spatially congruent or incongruent. It should be noted that Leo *et al*.^16^ reported a significant interaction of sound type and spatial congruency, but not a main effect of either factors. In other words, Leo *et al*.’s results were similar to our findings at 0 ms CTOA, namely that a looming sound or a spatially congruent sound does not, in itself, facilitate responses to a simultaneously presented visual target in the periphery—that is, not unless it is contrasted to performance in the opponent hemifield.

Why do looming sounds continue to confer a crossmodal cue benefit that persists until the end of their presentation, when static and receding sounds do not? The attentional mechanisms that underlie spatial orienting can be dichotomized into those that are early, involuntary, and transient, as opposed to those that are late, voluntary, and sustained^49^. From this perspective, looming sounds do not differ from other sounds insofar as they similarly support the early orienting of attention. However, our results suggest that looming sounds differ in terms of how they might counteract the withdrawal of early visuo-spatial attention, regardless of their loudness, perhaps by invoking late and voluntary processes. The current results demonstrate that looming sounds are different from receding and static sounds by conferring a late crossmodal cue benefit that is apparent at the end of their duration. Critically, this does not depend on their actual intensity, but rather how their intensity increases with time. The rest of this discussion will address the various explanations for why this is so.

The intuitive argument that looming sounds are ecologically salient and, hence, raise overall arousal cannot fully account for our current findings. If this was true, we would have expected response times to be faster whenever looming sounds accompanied visual targets. This might even have given rise to more false positives and, hence, lower sensitivity scores. Instead, our results demonstrate that looming sounds selectively differentiate from receding and static sounds with regards to how they confer a persistent cue benefit. Previous studies in multisensory integration have demonstrated that looming sounds can generally improve visual stimulus processing^16,38,39,44,45^. Nonetheless, our participants were not better at discriminating visual targets that appeared with the onset of looming sounds compared to when looming sounds preceded visual targets (see Fig. 1). Therefore, we are confident that our current findings relate to crossmodal influence of sounds on reorienting visuo-spatial attention, and not to multisensory integration. An argument for ecological saliency might also suggest stronger reflexive orienting. If this was true, looming sounds ought to have induced larger early cue benefits at the CTOAs of 250 ms. However, this does not appear to be true (see Fig. 1). In contrast, increasing the salience (i.e., intensity) of static sounds increased the magnitude of their late crossmodal cue benefit, but not for looming sounds (Experiment 2). Therefore, an ecological saliency account does not apply to our current findings even though we do not dispute that looming stimuli might still be preferentially processed because they: induce larger skin conductance responses^20^, elicit faster detection^39,50,51^, preferentially activate limbic^21^ as well as cortical^33^ structures, and are subjectively rated as being highly arousing^20^.

Looming sounds can be perceived as being louder than comparable static and receding sounds^25,26^. This is due to a recency effect, whereby observers respond to the sounds final instead of their overall intensity over time. However, Experiment 2 allows us to rule out this explanation, given that it manipulated cue intensity directly and found no difference in the late crossmodal cue benefits of soft and loud looming sounds. Therefore, we do not believe that the late crossmodal cue benefit of looming sounds is due to their perceived loudness.

Looming sounds can also be perceived as lasting longer than their receding equivalents^27–31^. To begin, the end of a receding sound may be suppressed because it is perceived as irrelevant reverberations of the sound rather than being part of the sound itself^52^. Alternatively, listeners might ignore the end of a receding sound given that they are expected to fade eventually^29^, unlike looming sounds that could rise indefinitely depending on their source intensity. Certainly, receding cues that are perceived as ending earlier can be expected to motivate earlier shifts of reoriented attention back to the manual tracking task. Nonetheless, it is unclear if this explanation can be directly applied to the duration perceptions of static and looming sounds. In fact, previous work have reported the perceived duration of looming sounds to be equivalent to static sounds^31^ or even shorter^29^. To the best of our knowledge, the perceived durations of looming sounds have yet to be reported as being longer than static sounds.

Finally, we could explain our current findings in terms of the anticipation that looming sounds might elicit in our observers for an approaching object, as long as the looming sound persists. In contrast, receding sounds signal a departing object and static sounds, a stationary object. Looming sounds provide information about object motion. More specifically, they indicate the intrusion of objects into the immediate space surrounding one’s body (i.e., peripersonal space; PPS). Previous work has shown that approaching sounds receive even more attention when entering one’s PPS than when approaching at further distance^53^. This could also explain why auditory looming cues in our study might support late mechanisms of attention orienting, while not differing from other cues with regards to early mechanisms. Looming sounds that continue to increase in intensity are more likely to indicate greater personal relevance. Furthermore, previous work has also found that looming objects can extend our perceived PPS boundaries^54,55^ and, in doing so, preserve a larger margin of safety around the body. This selective concern for looming objects could explain why looming sounds receive attention as long as they continue to indicate approach.

As long as a sound increases in intensity, it is suggestive of the time when an approaching object will appear^56,57^. Although intensity oftentimes determines stimulus saliency, its influence on attention is likely to be transient if it remains unchanged. Electrophysiological research in non-human primates have shown that looming sounds not only affect the primary auditory cortex, but also those areas that are involved in space recognition, auditory motion perception, as well as attention^33,58^. Similarly, fMRI studies have also implicated a network of regions that are selective to looming sounds that are involved in the evaluation of complex object motion (i.e., superior and middle temporal sulcus)^21,32^. More recently, an MEG study has shown sustained neural activity in the right temporo-occipito-parietal junction and bilateral inferior temporal gyrus, which tracked the increasing intensity of looming sounds with relatively long durations (i.e., 1600 ms). This resulted in significant differences against the neural activity generated by falling intensities of receding sounds, particularly at late periods (i.e., 900–1400 ms) after sound onsets. More interesting, these regions are not considered to be part of the auditory cortex, hence suggesting a supramodal influence of looming sounds on attention^34^. Sustained activity in these regions suggest that looming sounds exert a continuous influence on attention, as long as they continue to increase in intensity and indicate the potential approach of a relevant object.

The explanation that approaching objects sustain reoriented attention does not contradict an account of ecological saliency. Instead, it suggests that looming sounds can target later voluntary mechanisms of attention, besides early reflexive mechanisms. Indeed, our results indicate that looming sounds mitigate the withdrawal of early cuing benefits rather than enhance them. Therefore, our findings indicate that looming sounds oblige the visual system to continue paying attention to a region of the visual periphery that would otherwise remain neglected, especially when attentional resources are demanded elsewhere. In this regard, we believe that our results depended on the fact that we employed a manual tracking task that constantly demanded visuo-spatial attention^59^, thus exacting a significant cost whenever attention was directed elsewhere.

Our current findings inform the debate on whether motion stimuli are preferentially attended to because of their early motion onset^60–62^ or their motion properties (i.e., directionality^63,64^). The current results can be interpreted in favor of the latter. If stimulus change (or motion onset) was the primary driver of attentional shifts, we might even expect receding sounds to exert a larger early cuing benefit, given that they decreased in intensity exponentially. Instead, we find that the cuing benefits of looming sounds discriminate themselves towards the end of their presentation, namely when they are perceived as being closest to the observer and, hence, most relevant.

In conclusion, the current study reports that looming sounds exert a late crossmodal benefit on visuo-spatial attention that is apparent throughout their presentation. This is in spite of the demands for visuo-spatial attention by a central manual tracking task. Hence, this study extends the role of auditory looming from multisensory integration and highlights the role of auditory looming in capturing and reorienting attention away from a primary task. The important peculiarity of auditory looming in this context is the rising intensity which, independent of its overall intensity, compels observers to persist in attending to the cued location. After all, the gentle prowl of a tiger can be as deadly as the clumsy stampede of cattle, but only when they near us.

## Methods

### Participants

Thirty healthy volunteers participated in the current study (*Experiment 1*: 7 males, 8 females; mean age = 26.67 years ±4.78 s.d.; *Experiment 2*: 5 males, 10 females; mean age = 24.67 years ±3.79 s.d.). All participants reported normal hearing, normal (or corrected-to-normal) vision, and no history of neurological problems. They received written instructions, gave informed signed consent, and were remunerated 8 Euros/hour for their voluntary participation. The experimental procedure was approved by the Ethics Council at the University Hospital Tuebingen and carried out in accordance with their specified guidelines and regulations (see DOI 10.17605/OSF.IO/4WYGJ).

### Design

Experiments 1 and 2 employed a full factorial repeated measures design. Experiment 1 had two independent variables: (1) *CTOA* between an auditory cue and the peripheral visual target with three levels (either 0, 250, or 500 ms), (2) the intensity profile of the *Auditory Cue* with four levels (none, static, looming, and receding). Experiment 2 had two independent variables with two levels each: (1) intensity profile of the *Auditory Cue* (static, looming), (2) *Intensity* of the auditory cue (low, high). The *CTOA* in Experiment 2 was fixed at 500 ms. The primary dependent variable was the median response time for correct responses to the peripheral target.

Every session consisted of several 4.5 mins blocks of continuous manual tracking with 60 trials of a single-stimulus forced-choice (1AFC) peripheral tilt-discrimination task. Experiment 1 consisted of three sessions (15 blocks each) performed over consecutive days. *CTOA* was fixed for each block and the presentation order of *CTOA* was counterbalanced within sessions and across participants. Experiment 2 was conducted in one session (i.e., 20 blocks) on a single day.

### Stimuli

For Experiment 1, the auditory cues were 400 Hz tones with triangular^44^ complex waveforms, created using the MATLAB sawtooth function sampled at 44.1 kHz. Their duration was 500 ms and their intensity over time was shaped to assume one of three profiles: looming, receding, or static. The looming sound was characterized by a dynamic increase from 44 to 68 dB SPL, as measured at the participant’ ear position using an SPL meter [Brüel & Kjaer, Type 2238]. This change in loudness could be described as an audio object that approaches at the speed of 29 m/s towards the observer, with a time-to-contact of 500 ms^36^. The receding sound was created by reversing the looming sound in time. The static sound had a steady-state intensity that was the mean intensity level of the looming (and receding) sound, i.e. 56 dB. To avoid clicking noise at sound on- and offsets, all sounds were convolved with a trapezoid grating such that they had 5 ms ramps at the sound on- and offset.

**Figure 3:**
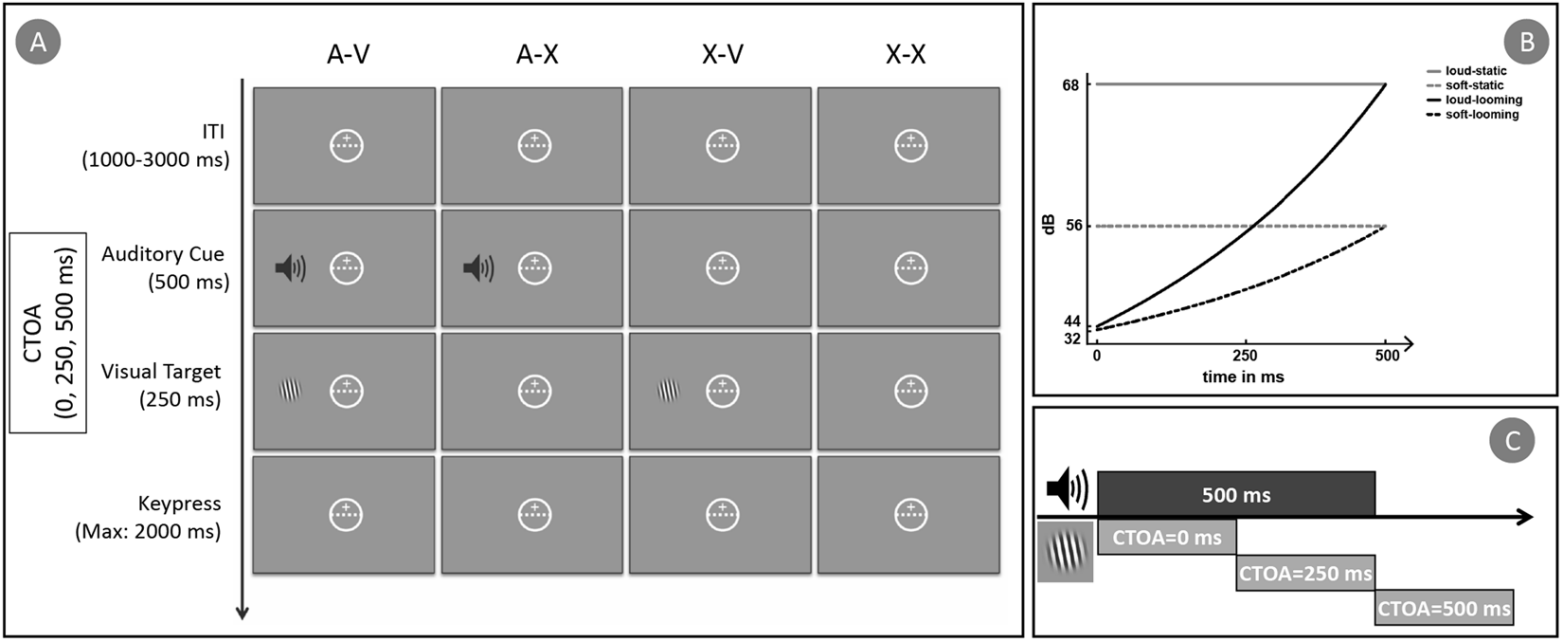
Experiment procedure and stimuli. (**A**) Four instances of trials of equal probability that could require participants to perform tilt-discrimination on a peripheral visual target (depicted larger than actual, for visibility). (**B**) Auditory cues used in Experiment 2, whereby the soft-static cue and loud-looming cue were the static and looming cue of Experiment 1. (**C**) Visual targets could appear at the onset of the auditory cue or after the onset.

For Experiment 2, the static and looming sounds were modified to create versions that ended with comparable low and high intensities. In Experiment 2, the original looming sound was regarded as the loud-looming cue and the original static sound as the soft-static cue. Accordingly, loud-static cue was the static sound with an adjusted intensity that matched the loud-looming cue’s end-intensity of 68 dB, while a soft-looming cue was a looming sound that began ended with an intensity of the original static sound 56 dB and began with an intensity of 32 dB. These intensity profiles are visualized in Fig. 3B. All auditory stimuli can be accessed at DOI 10.17605/OSF.IO/4WYGJ.

In both experiments, the peripheral visual targets were Gabor patches of 2 degrees visual angle in diameter, with a spatial frequency of 3.1 cycles/degree and a contrast of 50% (background gray = 20.3 cd/m^2^, Gabor patch black = 1.3 cd/m^2^, Gabor patch white 38.3 cd/m^2^). They were always presented 9° to the left or right of the manually tracked crosshair for 250 ms. A pre-testing adaptive procedure tuned the orientation tilts to be 70% orientation discrimination threshold of each participant (mean threshold and standard error for the left hemifield: 1.74° ± 0.27, and the right hemifield: 1.70° ± 0.21)^65^.

A compensatory visuo-motor tracking task, presented in the center, had to be performed continuously. A crosshair cursor comprising a vertical and horizontal line (0.70° long) was continuously and vertically displaced from a dotted horizontal reference line (5.43° long), along the vertical screen center. Participants rejected this cursor displacement to stabilize this cursor by deflecting a joystick forwards and backwards, which controlled the cursor’s vertical velocity and acceleration with equal weighting. In the absence of manual inputs, the cursor displacement was controlled by a quasi-random reference signal that was a sinusoidal function comprised of a sum of ten, non-harmonically related sine waves. This function had a variance of 1.62°^66^.

### Apparatus

The experiment was controlled with custom-written software in MATLAB 8.2.0.701 (R2013b) and Psychophysics Toolbox 3.0.12^67–69^). A ViewPixx Screen (60.5 × 36.3 resolution; 120 *H*z) presented all visual stimuli, at a fixed distance of 45 cm from chin-rest. Sound presentation was controlled by an ASIO compatible sound card (SoundBlaster ZxR; Creative Labs) and presented monophonically through either the left or right speaker of a pair of headphones (MDR-CD380; Sony). The right and left arrow inputs of a standardized keyboard were used for collecting left and right tilt discrimination responses respectively. A right-handed control stick (Hotas Warthog Flight Stick) was used for the central manual tracking task.

### Procedure

Prior to testing and after experiment briefing, participants performed five practice blocks of manual tracking only, followed by an adaptive procedure on a 1AFC tilt-discrimination task on peripheral visual targets^65^. The adaptive procedure determined the tilt that corresponded to the participant’s 70% orientation discrimination threshold. Participants fixated a static central cross throughout this adaptive procedure. To determine individual thresholds, we employed a 1-up-2-down staircase procedure with six interleaved staircases, evenly divided for the left and right hemifields. The vertical tilt of the Gabor stimuli had starting values of 0.0°, 2.5°, and 5.0° for three staircases per hemifield. Each staircase allowed for a maximum of 100 trials or terminated after 19 reversals, whichever came first. The first four reversals had the respective step sizes of 1.0°, 0.5°, 0.25°, and 0.1°, which then remained constant for the rest of the adaptive procedure. This was always performed in the first experimental session.

Upon completion, participants were allowed to perform the test blocks (Experiment 1: n = 45; Experiment 2: n = 20). Participants were required to perform two concurrent tasks on every test block: (1) a compensatory manual tracking task on a central crosshair, (2) a 1AFC tilt-discrimination task on peripheral visual targets. Mandatory 1.5 min rest breaks were provided between blocks.

In the compensatory tracking task, participants deflected the right-handed joystick in either the forwards or backwards direction to their body in order to counteract movements of the crosshair in either the upward or downward direction respectively. The goal was to stabilize a central crosshair on a horizontal dotted line. In the tilt-discrimination task, participants had to determine the tilt of peripheral targets when they appeared on either the left or right side of the crosshair. They responded by using their left index or ring finger to respectively indicate a left or right diagonal tilt. Participants were instructed to maintain fixation of the central crosshair of the manual tracking task throughout the experiment.

Trials occurred every 2000 ms ± 1000 ms (uniform distribution) and presented either an auditory cue only (A-X), a peripheral visual target only (X-V), an auditory cue followed by a peripheral visual target (A-V), or neither cue nor target (X-X). This ensured that the auditory cue was non-predictive of target appearance. When an auditory cue was presented, they were always presented via the headphone (i.e., right/left) that was on the same side as the upcoming visual target. When visual targets were presented, they appeared equally often on the left and the right of the central visuo-motor tracking task. In Experiment 1, they could occur at the onset of an auditory cue (if any), or 250 ms or 500 ms after the cue onset (see Fig. 3C). In Experiment 2, visual targets always appeared 500 ms after the auditory onset. A fixed duration of 2000 ms for keypress responses always took place after a visual target was supposed to be presented. The timelines of these four possible trials are illustrated in Fig. 3A.

After completing the required number of test blocks, participants were debriefed on the purpose of the experiment.

## Data Availability

The datasets generated during and/or analyzed during the current study are available in the OSF repository, 10.17605/OSF.IO/4WYGJ.
